# Small Islands, Small Ponds, Small Communities—Water Beetles and Water Boatmen in the Faroe Islands

**DOI:** 10.3390/insects13100923

**Published:** 2022-10-12

**Authors:** Leivur Janus Hansen, Agnes-Katharina Kreiling

**Affiliations:** Department of Terrestrial Zoology, Faroe Islands National Museum, Kúrdalsvegur 15, 188 Hoyvík, Faroe Islands

**Keywords:** aquatic coleoptera, Dytiscidae, Haliplidae, Corixidae, species richness, pond size, island biogeography, Faroe Islands, freshwater

## Abstract

**Simple Summary:**

The Faroe Islands in the North Atlantic consist of several small islands, and numerous small freshwater ponds can be found on most of them. These ponds are inhabited by species-poor aquatic communities. Faroese ponds are thus simple systems which lend themselves well to studying diversity of water insects and their interactions with the environment, which ultimately contributes to our understanding of patterns of biodiversity. In this study, we systematically collected water beetles and water boatmen from 57 ponds on the islands Streymoy and Eysturoy to obtain information on species distributions, diversity, and community composition, as well as their relationship with habitat characteristics. We found six small species of Dytiscidae and Haliplidae (Coleoptera) and two species of Corixidae (Heteroptera). There was a higher species diversity in shallower ponds, and community composition differed according to pond size. Geographical location and isolation between ponds did not influence diversity and community composition. We thus conclude that the distribution of water beetles and water boatmen in the Faroes is explained by habitat characteristics, specifically size and depth of the pond.

**Abstract:**

Water beetles of the families Dytiscidae and Haliplidae (Coleoptera) as well as water boatmen (Heteroptera: Corixidae) are well-studied groups in Northern Europe. In the Faroe Islands, their diversity is much lower than in the British Isles and Fennoscandia. Here, we first describe the communities of water beetles and water boatmen in Faroese ponds and, secondly, assess whether community compositions are driven by habitat characteristics or dispersal abilities of species. To this end, we sampled 57 ponds, ranging between <50 m^2^ and >50,000 m^2^. Environmental variables such as pond size, temperature, pH, and depth were measured, and distance to nearest neighboring pond was calculated as a measure of isolation. The sampling yielded 1522 individuals of eight species, with species richness of the ponds ranging between zero and six. Pond size (shoreline length) did explain differences in community composition, whereas water depth explained differences in diversity indices with lower diversity in deeper ponds. We found species-specific relations between abundance and shoreline length, e.g., *H. fulvus* and *H. palustris* being restricted to larger ponds. Lastly, water beetle and water boatmen communities in Faroese ponds are discussed in the light of island biogeography and species distributions in the North Atlantic islands.

## 1. Introduction

The interplay between dispersal ability, environmental tolerance, and intra- and interspecific interactions determines the distribution of species and, depending on available resources (biotic and abiotic), abundance and species richness at various spatial and temporal scales. To work on geographical ecology is to search for patterns [1], and it is often assumed to be advantageous to look at relatively simple systems, e.g., areas with few species and extreme environmental conditions, such as the North Atlantic islands.

The North Atlantic islands, from the British Isles and northwards, represent a gradient of increasing isolation (distance from source area) and environmental harshness, and many groups of land and freshwater species decrease in species richness along this gradient [2]. The Faroe Islands (hereafter referred to as the Faroes), located between Iceland and the Shetlands, are in a central position along this gradient of isolation. The aquatic insect fauna of the Faroes is characterized by the complete absence of Odonata, Ephemeroptera, and Plecoptera [3]. Although fish, mainly brown trout (*Salmo trutta* L.) and three-spined stickleback (*Gasterosteus aculeatus* L.), are present in some of the larger ponds, Coleoptera and Corixidae often are the top predators. Species richness of water beetles and water boatmen is much lower than in the British Isles and Fennoscandia [2]. Whether the species richness in the Faroes is restricted by the environment or by distance to the mainland has been the subject of some discussion [2,4,5,6].

In the Faroes, water beetles and water boatmen have mainly been recorded as part of wider collection effort without targeting these species. Only few ecological studies have been conducted of inland waters in the Faroes. These studies were either concentrated on one lake or on a restricted part of the ecosystem, such as a seasonal study of the benthic community in Leynavatn and analyses of phytoplankton community composition in eleven lakes [7]. Brown [8], however, conducted an extensive study on water boatmen in which he also described several types of water bodies in the Faroes. Foster and Hansen [9] re-examined existing records of water beetles and also made an extensive survey. Hansen and Foster [10] published an updated identification of the water beetle *Elodes pseudominuta* Klausnitzer, previously recorded as *Elodes minuta* (Linnaeus).

Water beetles of the families Haliplidae and Dytiscidae (Coleoptera) and water boatmen (Heteroptera: Corixidae) are trophically similar and can be considered a discrete ecological guild [11]. They are thus often studied together, and much is known about their distribution and ecology in northwestern Europe [12,13,14]. A high proportion of water beetle and water boatmen species possess good flight abilities [12,15,16,17]. Many species are spatially distributed in patchy networks of ponds with interacting populations that are subject to extinctions and recolonizations, i.e., metapopulations [18]. Studies of water beetles have demonstrated relationships between species richness and community composition and various habitat characteristics, e.g., pond size and productivity [19,20,21], acidity [22,23], salinity [24,25], degree of exposure to wave stress [26], vegetation structure [26,27,28], duration of inundation [29,30], seasonal change [31,32], and distance between ephemeral habitats and permanent source areas [30]. As suggested by Ribera et al. [33], habitat effects on aquatic beetles may impose constraints at a local level, which may determine species richness at larger temporal and spatial scales. Furthermore, Eyre et al. [34] demonstrated that the distribution of water beetle species is closely related to climatic variables and may be used for monitoring climatic change.

Our first (I) objective was to describe the water beetle and water boatmen communities in ponds and lakes in the Faroes.

Our second (II) objective was to determine whether those communities are shaped by habitat characteristics (environmental factors) or as a result of stochastic distribution events (geographical location and isolation).

We hypothesized that the species which have successfully colonized the islands are characterized by good dispersal ability and hardiness in terms of environmental tolerance. As a consequence, we expected a high degree of geographical and ecological overlap between the species. Thus, patterns in distribution, species richness, and community composition should be strongly affected by environmental variables such as pH, pond size, water temperature, and altitude.

Lastly, and assuming that communities are shaped by environmental variables, our third (III) objective was to determine the relative importance of these on species diversity.

Hereby, we hypothesized that pond size is an important variable, as shown by [19,20,21]. As our sampling design restricted us to collect water beetles and water boatmen from the littoral zone, we use shoreline length (pond perimeter) as a variable instead of the correlated variable pond size.

## 2. Materials and Methods

The Faroes consist of 18 islands and several islets situated in the North Atlantic (approx. 62° N and 7° W) about 675 km W of Norway, 300 km NW of Shetland, and 450 km SE of Iceland. The total land area is 1396 km^2^ [35]. The climate is oceanic with frequent and strong winds (predominantly from W and SW), much rainfall, mild winters, and cool summers, with mean temperatures of +4 °C and +11 °C, respectively.

A total of 2293 waterbodies with a surface area of more than 100 m^2^ have been identified and mapped, including some smaller ones [36,37]. The majority of the identified ponds were smaller than 1000 m^2^, and only 28 waterbodies had a surface area larger than 50,000 m^2^. Those, and some which are smaller and deeper, can be defined as lakes, according to Richardson et al. [38]; however, for the sake of brevity, all studied waterbodies will be referred to as ponds in this paper.

Being heavily influenced by surface runoff, ponds and streams in the Faroes differ from continental waterbodies in permanency and water level fluctuations. In the Faroes, smaller ponds and streams tend to dry out from time to time during summer, and others fluctuate considerably in size.

**Figure 1:**
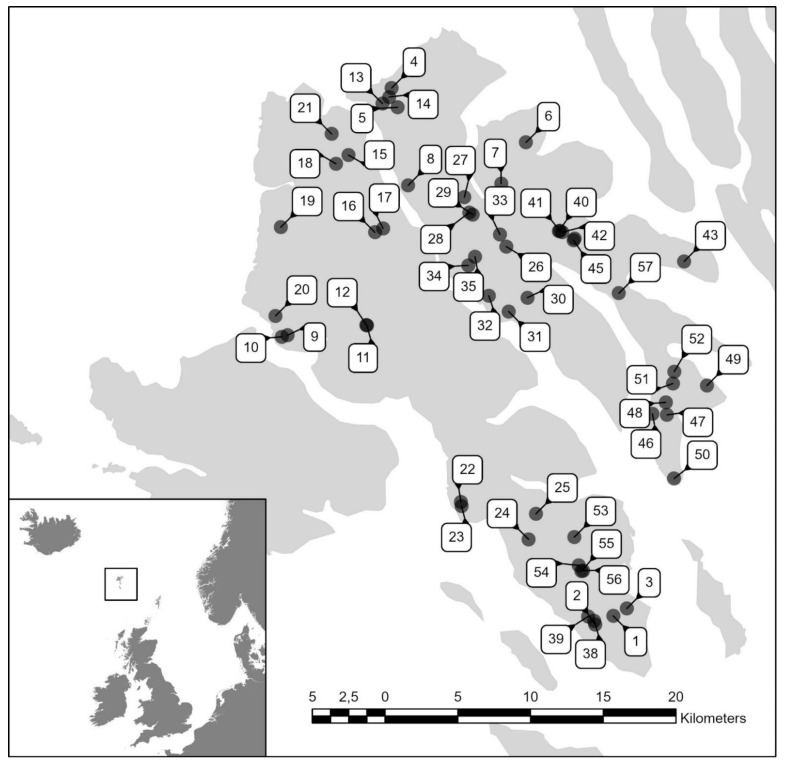
Map of the 57 sampled ponds on the islands Streymoy and Eysturoy. The numbers refer to ponds, as listed in Table 1. The figure inset shows the location of the Faroe Islands in the North Atlantic between Iceland, the British Isles, and Norway.

All of the 2293 mapped ponds were grouped into five size classes (<100 m^2^, 100–999 m^2^, 1000–9999 m^2^, 10,000–99,999 m^2^, and >100,000 m^2^). Of those, 57 ponds (Figure 1, Table 1) were selected for the study using a stratified random sampling design, which ensured that ponds of different size classes were distributed throughout the duration of fieldwork and geographically over the sampled areas. Fieldwork took place between 6 May and 8 June 2002.

At each pond, 20 samples were taken at random intervals along the perimeter and between 0 and 2 m from the shoreline. These points were produced using a random number generator.

The sampling technique was based on Nilsson [39]. With a circular sweep net (0.25 m in diameter) with 1 mm mesh size, a portion of the water was filtered. The time used for filtering (net sweeping) was a minimum of 2 min. If the bottom was clean gravel with good visibility, the time used sweeping was usually shorter than if the bottom was muddy or had a vegetation cover (and thereby clogged the net). When debris clogged the net, it was emptied into a white flat tray which contained some water, and net sweeping was resumed until the sampling point subjectively was considered sufficiently worked. The remaining material in the net was placed into the same tray, and water beetles and water boatmen were collected, killed, and preserved in 70% ethanol. To ensure a constant sampling effort, a 0.5 × 0.5 m square metal frame was used, roughly within which sampling occurred. Species of water beetles were identified according to Holmen [12] and Nilsson and Holmen [14], and water boatmen according to Jansson [13]. In addition, specimens from the Museum of Zoology in Lund were used as references. The nomenclature follows Nilsson [40]. Some specimens from the present study were donated to the Museum of Zoology in Lund, but the bulk of the material is stored in the Faroe Islands National Museum (FOMNH).

In addition, we also recorded pH and water temperature close to the surface (<0.2 m depth), and to avoid any possible stratification effects, the water samples for pH and temperature measurements were taken at a distance from the bank where the pool surface was assumed to be unprotected from wind. The depth of the ponds was measured in the deepest part of the sampled area, and type of bottom substrate and amount of aquatic vegetation were estimated and noted. Time constraints did not permit us to ascertain presence of fish (trout and stickleback) in a systematic manner, but casual observations of fish were noted in ten ponds (with trout in six ponds, stickleback in three, and both in one). In addition, altitude, longitude, latitude, pond size, and shoreline length (pond perimeter) were obtained through the GIS software Mapinfo 6.5 [41]. As a measure of isolation between ponds, we calculated the planar distance to the closest pond using Generate Near Table in ArcGIS Pro [42].

Statistical analyses were performed in R 4.2.1 [43].

As a measure of alpha diversity, we calculated species richness (N_0_), Shannon diversity (N_1_), and Shannon evenness (as E = N_0_/N_1_; Hill’s ratio) according to Borcard et al. [44] using the diversity function in the R package vegan [45].

Possible differences between those diversity indices from ponds on the two islands, Eysturoy and Streymoy, were tested with Welch’s two-sample *t*-test.

To assess the collinearity of the environmental variables, correlations between the environmental variables were calculated (function chart.Correlation in PerformanceAnalytics package) [46]. In cases of high collinearity, we retained the independent and most representative variables but excluded the others from subsequent tests, resulting in initial models with the following explanatory variables: island, distance from nearest neighboring pond as a measure of isolation between waterbodies, altitude, shoreline length, water depth, sediment depth, and water temperature.

The effects of geographical location (namely, island), water temperature, altitude, water depth, sediment depth, shoreline length, and distance from nearest neighboring pond (near_dist) (explanatory variables) on the community composition (response variables) were tested with a PERMANOVA (function adonis2 in the vegan package) and visualized with nonmetric multidimensional scaling (NMDS) using the functions *metaMDS* and envfit in the vegan package. Bray–Curtis was used as dissimilarity distance measure.

To evaluate the importance of shoreline length on species distributions, we plotted species abundances as well as diversity indices against log-transformed shoreline length. The dependence of species richness on environmental variables was analyzed with generalized linear models (function glm in stats package [43]) with the Poisson link function, whereas Shannon diversity and evenness were analyzed with multiple linear regressions (function lm in stats package). Initial models were simplified by a stepwise procedure, excluding the least significant variable in each round until the minimal adequate model was reached. Model selection was based on the results of ANOVA tests, to assess whether the models differed significantly, as well as the Akaike information criterion (AIC).

## 3. Results

A total of 1522 individuals (1246 water beetles and 276 water boatmen) were found in this study, belonging to eight different species: *Arctocorisa carinata* (C. Sahlberg), *Callicorixa wollastoni* (Douglas & Scott), *Haliplus fulvus* (Fabricius), *Hydroporus pubescens* (Gyllenhal), *Hydroporus erythrocephalus* (Linnaeus), *Hydroporus palustris* (Linnaeus), *Boreonectes multilineatus* (Falkenström), and *Agabus bipustulatus* (Linnaeus). Two additional species of Dytiscidae, *Hydroporus nigrita* (Fabricius) and *Hydroporus memnonius* Nicolai, have previously been recorded in the Faroes [8,47] but were not found in our study.

Local species richness varied between 0 and 6 (Table 1). There were no differences between the ponds on the islands Eysturoy and Streymoy in mean local species richness (Welch’s two-sample *t*-test: t = 0.8729, df = 54.22, *p* = 0.387), Shannon diversity (Welch’s two-sample *t*-test: t = 0.5975, df = 55, *p*-value = 0.553), and Shannon evenness (Welch’s two-sample *t*-test: t = 0.9816, df = 40.15, *p*-value = 0.332). However, *H. fulvus* only occurred in ponds on Eysturoy.

**Figure 2:**
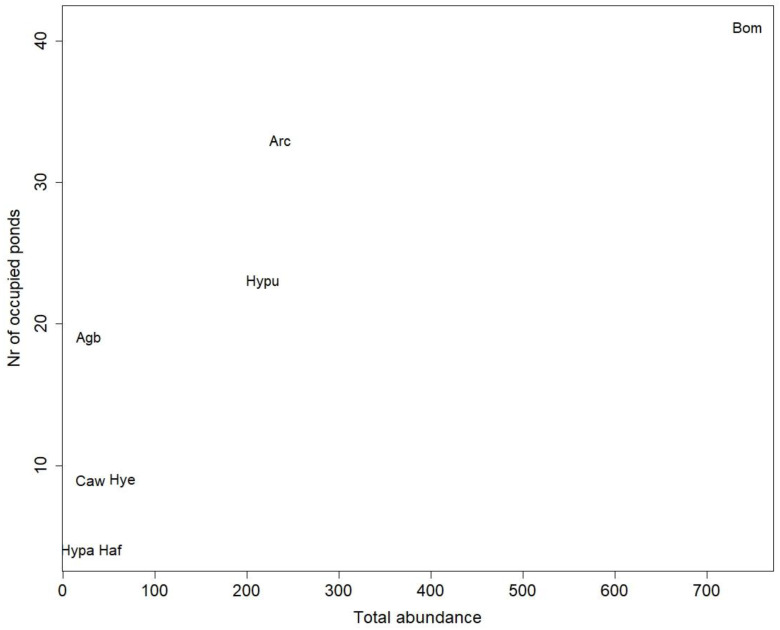
Frequency and abundance of water beetles and water boatmen in Faroese ponds. Species acronyms are as listed in Table 1.

There was a positive relationship between abundance and number of ponds occupied (frequency), with *B. multilineatus* as the most abundant and by far the most widely distributed species (Figure 2). The two species *A. carinata* and *H. pubescens* had almost equal abundances but the former occupied more ponds. For the remaining five species, with relatively low abundances, the number of ponds occupied ranged between 4 and 19. Species occurring frequently in ponds (i.e., *B. multilineatus* and *A. carinata*) co-occurred with all other species, and species with a low frequency (*H. palustris* and *H. fulvus*) co-occurred with few of the other species.

**Figure 3:**
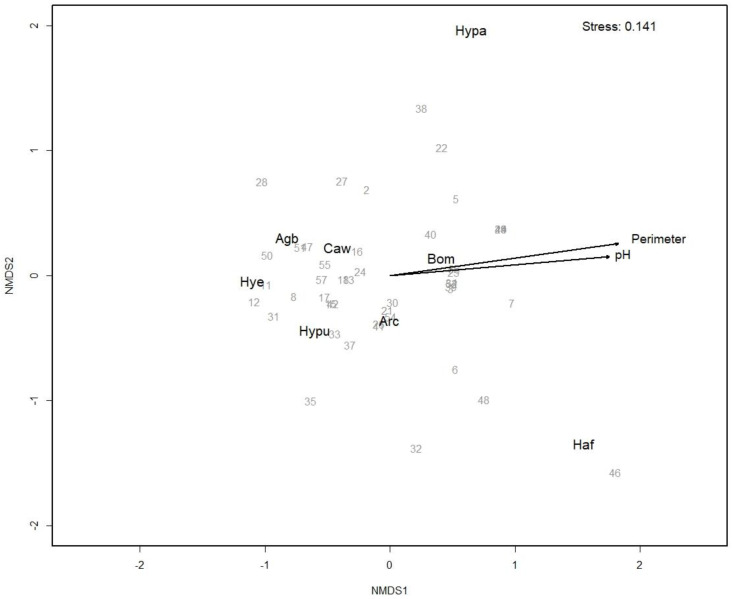
Community composition of water beetles and water boatmen in Faroese ponds. Nonmetric multidimensional scaling of species (black text) and ponds (grey numbers). Significant associations (*p* < 0.05) of the environmental variables to the axes in the envfit analysis are shown with arrows. Pond numbers and species acronyms are as given in Table 1.

Community composition of water beetles and water boatmen seemed to be shaped by environmental variables rather than geography or isolation between water bodies, with shoreline length (pond perimeter) and water depth being significant explanatory variables (Figure 3, Table 2). *B. multilineatus*, *H. fulvus*, and *H. palustris* were associated with ponds with a longer shoreline, whereas the other five species tended to be found in smaller ponds (Figure 3). Due to its correlation with shoreline length, pH was not included in the model but was visualized in the ordination as its association with the axes (Figure 3).

**Figure 4:**
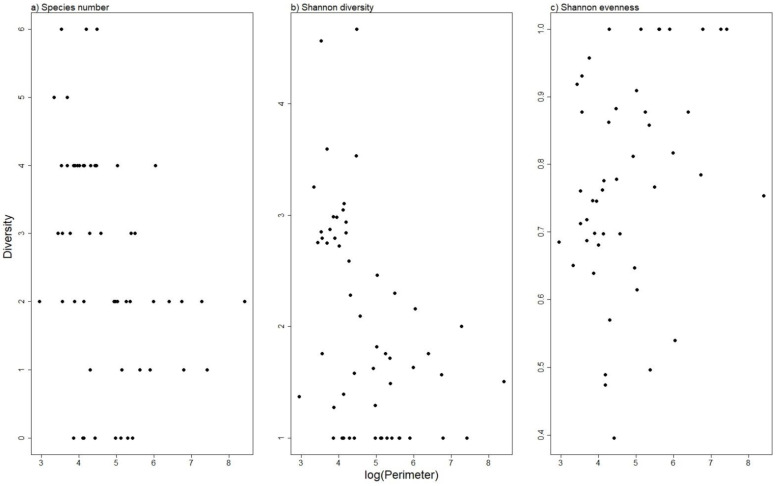
Relationship between diversity and shoreline length in aquatic beetle communities in Faroese ponds. Species richness (**a**), Shannon diversity (**b**), and Shannon evenness (**c**) are shown in relation to log-transformed shoreline length (pond perimeter).

Species number and Shannon diversity were highest in the smaller ponds (Figure 4a,b), whereas evenness seemed higher in larger ponds (Figure 4c). Shoreline length was, however, not significant in the linear models, unlike water depth, which proved a significant variable for all three diversity indices (Table 3). Species number and Shannon diversity were lower in deeper ponds, whereas evenness was higher (i.e., species composition more even) in deeper ponds.

**Figure 5:**
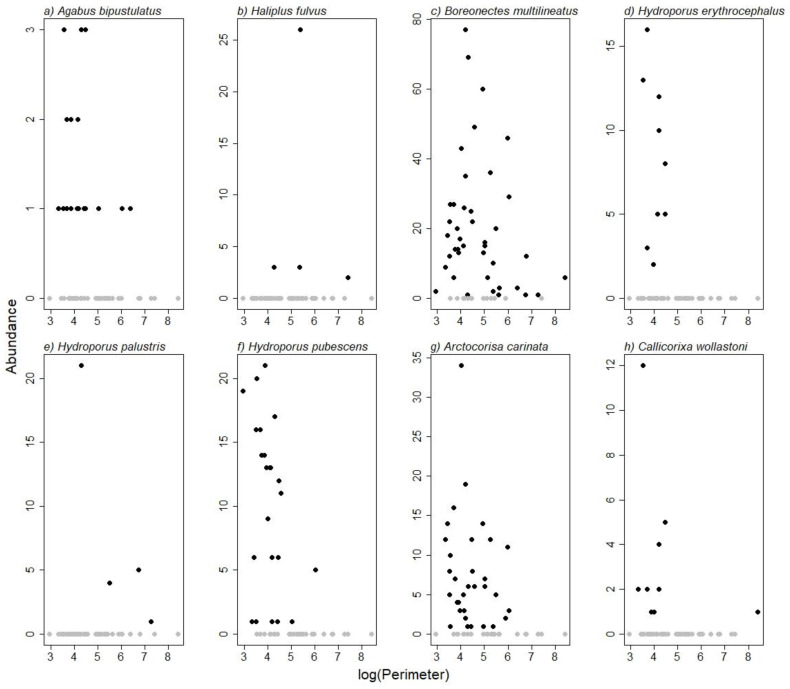
Species-specific relations to pond size. Abundance of water beetles (**a**–**f**) and water boatmen (**g**,**h**) in relation to log-transformed shoreline length (perimeter) of the ponds. Ponds in which the species were present are represented as black dots, and ponds in which the species were absent as grey dots.

There were species-specific relations to shoreline length (Figure 5). *H. fulvus* and *H. palustris* only occurred in ponds with a longer shoreline, *B. multilineatus* was fairly evenly distributed over ponds with varying shoreline lengths, and the remaining five species were mainly found in ponds with shorter shoreline (Figure 5).

## 4. Discussion

Our first (I) objective of the study was to describe the community of water beetles and water boatmen in Faroese ponds. Species richness was low, with only eight species found of ten previously reported species. On average, there were two to three species per pond, and all but one species (*H. fulvus*) were geographically widely distributed. This is in line with previous reports of species poverty of water beetles and water boatmen [4,8,47] and a variety of other taxa in the Faroes [48,49].

Our second (II) objective was to determine whether water beetle and water boatmen communities were shaped by habitat characteristics of the ponds (environmental variables) or the result of different dispersal abilities of the species (proxied by geographical location and isolation between water bodies). We had predicted that environmental variables were more important drivers of community composition than variables related to dispersal abilities, and this was supported by our results. Shoreline length (a proxy for pond size) and water depth were the significant variables in explaining the community composition in the ponds.

Lastly (III), we wanted to determine the effects of those variables on species diversity. We had predicted that diversity would be influenced by pond size, but that was not supported by our results. Water depth, but not shoreline length, was correlated with all three diversity indices.

In the British Isles, the total number of species of the three aquatic insect families included in this study, Haliplidae, Dytiscidae, and Corixidae, is 173 (19, 120, and 34, respectively) [17,50], of which at least 42 and 49 have been found in Shetland and Orkney, respectively [51,52]. In the Faroes, some 300 km north of Shetland, only 10 species have been recorded [8,9], and further north, in Iceland, 8 species have been reported [53]. Thus, species number decreases with latitude and distance from the most likely source areas (British Isles and Fennoscandia), and the species pool of the Faroes is small.

Presumably, Faroese species have arrived from the British Isles and Fennoscandia, but only a small fraction (5 and 3.5%, respectively) of the relevant species pools of these two source areas has succeeded in colonizing the Faroes. Considering the dispersal ability of many members of the family Dytiscidae, one would expect more species to have reached the islands than those presently occurring there. That at least some of the species apparently absent in the Faroes are capable of reaching remote islands is suggested by the fact that three of the five Dytiscidae species found in Iceland (*Nectoporus sanmarkii* (Sahlberg), *Agabus uliginosus* L. and *Colymbetes dolabratus* (Paykull)) have not been recorded in the Faroes [53]. On the other hand, *H. fulvus* occurs in both Iceland and the Faroes, although species of the family Haliplidae are claimed to have a relatively low dispersal ability [12]. Of the two Corixidae species occurring in the Faroes, only one, *A. carinata,* is also found in Iceland [53]. It is also noteworthy that the Faroese water beetle fauna includes no large *Dytiscus* L. or *Colymbetes* Clairville and that *A. bipustulatus* is the largest species (10–11 mm), whereas the others (including the two previously recorded species not found in present study) are all in the range 3.5–4.5 mm in size.

Even the highest number of species (six) found in any single pond is low compared to studies in other northern regions, e.g., 32 species of Dytiscidae found in a 500 m^2^ pond in northern Sweden [11] and 27 Coleoptera and nine Heteroptera species in bog pools in an area of 0.25 km^2^ in Newfoundland [54]. However, regional species pools and local species richness are, for instance, much higher at high latitudes in northern Sweden than further south in the Russian Far East [55]. Thus, latitude and harsh climatic conditions may not preclude more species to be present. Nevertheless, the climatic conditions in the Faroes may still be involved by causing the generally low productivity and diversity of the lentic waters [56], which certainly may be of relevance to the regional species richness. In addition, the oceanic climate with low summer temperatures and mild winters are the opposite of the conditions prevailing in, e.g., northern Fennoscandia. It could be hypothesized that the absence of low winter temperatures in the Faroes poses a problem to potential colonizers with life cycles adapted to a continental climate.

Environmental variables, namely, pond size (shoreline length) and water depth, shaped the composition of water beetle and water boatmen communities in Faroese ponds. This is in line with the findings of several studies in other parts of the world. In his study on aquatic beetle communities of ponds in Southern Germany, Flechtner [19] found pond size to affect communities, with Dytiscidae preferring smaller and Haliplidae larger ponds. Similarly, Dytiscidae communities in Finland differ along a gradient of water body size [21].

Naturally, ponds differ from each other in many ways and not only with respect to the variables measured in this study. The permanency of a pond and the predictability of changes are important factors influencing species composition [57,58,59]. As our study was conducted during a short period of time in late spring, we have no observations on the permanency and stability of the water bodies included. Ribera and Vogler [60] show that stability of the habitat exerts differential selection pressure, resulting in different types of inhabitants.

Another variable we did not measure systematically was predation pressure by fish, which was shown to be an important driver of aquatic communities [61,62,63]. During sampling, we noted fish presence (brown trout and stickleback) in ten of the 57 ponds in this study. Although the number of ponds with fish may turn out higher when properly investigated, it nevertheless seems that only a small proportion of Faroese ponds are inhabited by fish, and we thus feel confident to exclude fish predation as a possible factor deciding over community composition of water beetles and water boatmen in the Faroes as a whole.

Positive relationships between pond size and species richness have been documented [20]. In our study, species richness did not increase with pond size. However, most of our studied ponds were bigger than 100 m^2^—only one sampled pond was smaller than 50 m^2^—and we might have seen a positive relationship between pond size and species richness if we had included more smaller ponds into our sampling scheme. Nilsson and Svensson [64] state that species richness and abundance seem to peak in pond sizes between 10 and 100 m^2^. Peak abundances varied between species in our study. *H. fulvus* and *H. palustris* abundances peaked in ponds with a size of >1000 m^2^ and *B. multilineatus* abundance in ponds of 200–300 m^2^ in size, whereas the remaining five species were most abundant in ponds of sizes around 100 m^2^.

Diversity of water beetles and water boatmen was lower in deeper ponds, which is in agreement with the general rule of thumb stated by Nilsson [40] that “the less water the more beetles”. Pond depth could also be linked to steepness of pond margin, which was not a measured variable in this study but has since been found to affect water beetle communities [63]. Dytiscidae species richness and abundance in urban ponds in Finland were higher in ponds with shallow margins [63].

*Haliplus fulvus* and *Hydroporus palustris* were only encountered in the larger ponds, with low abundances and with few (1–3) other species present. Their occurrence in the larger ponds is consistent with observations in other regions of the world, where they are more confined to lakes than to ponds. Both these species have wide geographical distributions [12,14], which is the opposite of what to expect of species with a preference for stable habitats [60]. *B. multilineatus* exhibited the highest evenness of occupancy with respect to pond size, and by far the highest overall abundance and frequency, probably occurring all over the Faroes. This is consistent with the association between local abundance and regional distribution [20,65], and the fact that it is the most widespread species in the northwest of Europe [9]. The species assemblage of the present study displays this general pattern, though *A. bipustulatus* deviates from it, and *A. carinata* and *H. pubescens* had wider distributions than some species with similar mean abundances. However, the low abundance found in *A. bipustulatus* may be an underestimate as it has been reported that few *Agabus* individuals are obtained when net sampling is applied, as they stay hidden during the day [11] and the actual abundance is possibly considerably higher. We can think of no explanation as to why *H. fulvus*, *H. palustris*, and *H. erythrocephalus*, all with reasonably high local abundances, are not found more often, though it can be noted that two of them are “large-pond species”.

## Figures and Tables

**Table 1:** Geographical location, environmental variables, diversity indices, and species abundances of the sampled ponds. Temperature is given in °C, sediment depth (“Sed.depth”) and water depth (“Wat.depth”) in m, length of shoreline (“Perimeter”) in m, and altitude above sea level in m. The three different diversity indices used are species richness (“Spec.rich.”), Shannon diversity (“Shan.div.”), and Shannon evenness (“Evenness”). Number of water beetles and water boatmen in the pond samples are given, and the species are abbreviated with the following acronyms: Agb—*Agabus bipustulatus*, Arc—*Arctocorisa carinata*, Caw–*Callicorixa wollastoni*, Haf—*Haliplus fulvus*, Bom—*Boreonectes multilineatus*, Hye—*Hydroporus erythrocephalus*, Hypa—*Hydroporus palustris*, and Hypu—*Hydroporus pubescens*.

Pond ID	Latitude	Longitude	pH	Temperature	Sed. Depth	Wat. Depth	Perimeter	Dist. Pond	Altitude	Spec.rich.	Shan.div.	Evenness	Agb	Arc	Caw	Haf	Bom	Hye	Hypa	Hypu
1	61.9766	−6.7842	6.42	12.2	0.5	1.5	60	8	248	0	–	–	0	0	0	0	0	0	0	0
2	61.9716	−6.8081	5.87	19.9	0	0.3	48	94	320	2	1.28	0.64	1	0	0	0	14	0	0	0
3	61.9809	−6.7663	5.66	13.7	0.5	0.4	151	98	178	2	1.82	0.91	0	6	0	0	15	0	0	0
4	62.3064	−7.0540	6.01	18.2	0	0	83	43	162	0	–	–	0	0	0	0	0	0	0	0
5	62.2943	−7.0469	–	15	0	0.4	4492	537	193	2	1.51	0.75	0	0	1	0	6	0	0	0
6	62.2703	−6.8783	6.8	10.3	0	0.3	72	1305	270	3	2.59	0.86	0	1	0	3	1	0	0	0
7	62.2453	−6.9130	6.6	12.3	0	0.5	212	1905	353	2	1.72	0.86	0	0	0	3	10	0	0	0
8	62.2462	−7.0360	5.02	20.8	0.5	0.6	88	8	215	6	4.67	0.78	1	8	5	0	22	8	0	12
9	62.1558	−7.2010	7.01	13.9	0	1.5	226	341	249	0	–	–	0	0	0	0	0	0	0	0
10	62.1549	−7.2087	6.68	9	0.4	1	60	341	236	0	–	–	0	0	0	0	0	0	0	0
11	62.1604	−7.0961	5.07	10.3	0	0.5	87	18	364	4	3.53	0.88	3	12	0	0	0	5	0	6
12	62.1606	−7.0966	5.07	9.2	0	0.5	63	18	365	4	3.10	0.78	2	3	0	0	0	5	0	13
13	62.2969	−7.0668	5.94	16.9	0.05	0.2	420	15	190	4	2.16	0.54	1	3	0	0	29	0	0	5
14	62.3009	−7.0577	–	15	0	0.5	73	240	169	1	1	1	0	0	0	0	0	0	21	0
15	62.2659	−7.1139	6.13	18.1	0.2	0.2	61	33	334	4	3.05	0.76	1	5	0	0	15	0	0	13
16	62.2177	−7.0818	6.68	18.5	0.1	0.5	83	409	406	4	1.58	0.40	1	1	0	0	25	0	0	1
17	62.2200	−7.0709	–	0	0	0	47	117	340	4	2.99	0.75	2	4	0	0	20	0	0	14
18	62.2607	−7.1308	6.33	13.8	0.1	0.3	74	22	421	4	2.28	0.57	3	6	0	0	69	0	0	17
19	62.2225	−7.2061	–	7	0	0.4	47	22	487	0	–	–	0	0	0	0	0	0	0	0
20	62.168	−7.2162	6.73	10	0	1.5	62	461	168	0	–	–	0	0	0	0	0	0	0	0
21	62.2791	−7.1352	5.65	11.6	0.1	0.4	55	8	338	4	2.72	0.68	0	34	1	0	43	0	0	9
22	62.0498	−6.9791	–	11	0	1.5	1438	36	245	2	2	1	0	0	0	0	1	0	1	0
23	62.0474	−6.9785	6.48	13	0.2	0.5	274	36	247	1	1	1	0	0	0	0	1	0	0	0
24	62.0256	−6.8919	5.87	14.1	0	0.4	152	493	470	4	2.46	0.61	1	7	0	0	16	0	0	1
25	62.0412	−6.8813	6.29	11.9	0.05	0.2	62	95	340	2	1.39	0.70	0	3	0	0	26	0	0	0
26	62.2064	−6.9088	5.12	13.4	0.1	0.2	35	49	393	3	2.79	0.93	0	10	0	0	27	0	0	20
27	62.2377	−6.9622	6.71	9.6	0	1.5	599	128	324	2	1.75	0.88	1	0	0	0	3	0	0	0
28	62.2281	−6.9563	5.03	11.9	0.1	0.4	35	45	380	2	1.75	0.88	3	1	0	0	0	0	0	0
29	62.2268	−6.9518	6.61	12.4	0.3	0.4	198	112	338	0	–	–	0	0	0	0	0	0	0	0
30	62.1745	−6.8832	5.05	12.9	0.1	0.3	97	11	403	3	2.09	0.70	0	6	0	0	49	0	0	11
31	62.1664	−6.9085	6.11	18.3	0	0.1	40	13	232	4	2.75	0.69	1	0	0	0	6	3	0	16
32	62.1764	−6.9340	6.76	17.5	0.1	1	364	93	208	1	1	1	0	2	0	0	0	0	0	0
33	62.2140	−6.9168	-	10	0	0.1	52	65	218	4	2.98	0.75	0	3	0	0	17	2	0	13
34	62.1956	−6.9599	6.53	11.8	0	0.4	138	17	347	2	1.62	0.81	0	14	0	0	60	0	0	0
35	62.2008	−6.9506	5.56	12	0.4	0.4	19	59	406	2	1.37	0.68	0	0	0	0	2	0	0	19
36	61.986	−6.8317	6.53	16.2	0.2	0.5	226	46	205	0	–	–	0	0	0	0	0	0	0	0
37	61.9875	−6.8280	5.67	0	0	0.4	49	11	210	4	2.79	0.70	0	4	1	0	13	0	0	21
38	61.9740	−6.8093	6.25	14.4	0	1	842	123	326	2	1.57	0.78	0	0	0	0	1	0	5	0
39	61.9769	−6.8171	6.63	11.9	0.5	0.3	191	30	274	2	1.75	0.88	0	12	0	0	36	0	0	0
40	62.2149	−6.8386	-	5	0	0.4	243	16	497	3	2.30	0.77	0	5	0	0	20	0	4	0
41	62.2150	−6.8373	5.86	19.4	0.05	0.3	43	5	500	3	2.87	0.96	0	7	0	0	14	0	0	14
42	62.2141	−6.8343	-	10	0.2	0.3	34	28	490	4	2.85	0.71	1	5	0	0	22	0	0	16
43	62.1935	−6.6749	6.83	13.8	0.1	0.4	166	559	290	0	–	–	0	0	0	0	0	0	0	0
44	62.2100	−6.8181	6.7	13.2	0	1.5	883	29	366	1	1	1	0	0	0	0	12	0	0	0
45	62.2089	−6.8196	–	8	0	0	170	10	366	1	1	1	0	0	0	0	6	0	0	0
46	62.1004	−6.7236	6.7	13.9	0	1.5	1666	5	16	1	1	1	0	0	0	2	0	0	0	0
47	62.0994	−6.7045	6.23	20.1	0.05	0.3	40	112	144	5	3.59	0.72	2	16	2	0	27	16	0	0
48	62.1072	−6.7055	6.85	20.4	0	0.4	217	54	140	3	1.49	0.50	0	1	0	26	2	0	0	0
49	62.1166	−6.6505	–	10	0	0	276	67	191	1	1	1	0	0	0	0	3	0	0	0
50	62.0600	−6.6982	5.48	15.7	0.1	0.3	34	16	106	6	4.56	0.76	1	8	12	0	12	13	0	1
51	62.1186	−6.6953	4.81	12.9	0	0.5	66	203	243	6	2.93	0.49	1	2	4	0	35	12	0	1
52	62.1259	−6.6930	6.68	11.4	0.2	0.3	399	172	206	2	1.63	0.82	0	11	0	0	46	0	0	0
53	62.0260	−6.8318	5.9	12.7	0	1	144	47	316	0	–	–	0	0	0	0	0	0	0	0
54	62.0085	−6.8273	5.34	3.4	0	0.4	31	35	193	3	2.75	0.92	0	14	0	0	18	0	0	6
55	62.0049	−6.8238	6.72	14.8	0.2	0.4	28	15	181	5	3.25	0.65	1	12	2	0	9	0	0	1
56	62.0051	−6.8216	-	17	0.2	0.3	144	10	180	2	1.29	0.65	0	1	0	0	13	0	0	0
57	62.1753	−6.7622	5.26	12.3	0	0.25	66	1305	116	6	2.84	0.47	1	19	2	0	77	10	0	6

**Table 2:** Dependence of the community composition on significant environmental variables according to the PERMANOVA. “F Model” indicates variation within the samples, and “r^2^” shows the percentage of the explained variance.

Variable	F Model	r^2^	*p*-Value
Perimeter	2.936	0.060	0.012
Water depth	2.350	0.048	0.033

**Table 3:** Dependence of diversity indices on environmental variables. The adjusted r^2^ value shows the variance explained by the linear models. Slope (b), standard error (SE), *t*-values, and *p*-values are shown for variables in the minimal adequate models for species richness, Shannon diversity, and Shannon evenness.

Diversity Index	Adjusted r^2^	Variable	b	SE	*t*-Value	*p*-Value
Species richness	-	Temperature	−0.013	0.015	−0.838	0.402
		Water depth	−0.009	0.003	−3.203	0.002
Shannon diversity	0.139	Temperature	−0.018	0.011	−1.617	0.111
		Water depth	−0.004	0.001	−2.858	0.006
Evenness	0.093	Temperature	−0.008	0.006	−1.348	0.185
		Water depth	0.002	0.001	2.297	0.027

## Data Availability

The data presented in this study are available on request from the corresponding author.
